# Optical Spring Effect in Micro-Bubble Resonators and Its Application for the Effective Mass Measurement of Optomechanical Resonant Mode

**DOI:** 10.3390/s17102256

**Published:** 2017-09-30

**Authors:** Zhenmin Chen, Xiang Wu, Liying Liu, Lei Xu

**Affiliations:** 1 Key Lab for Micro and Nanophotonic Structures (Ministry of Education), Department of Optical Science and Engineering, School of Information Science and Engineering, Fudan University, Shanghai 200433, China; 12110720020@fudan.edu.cn (Z.C.); wuxiang@fudan.edu.cn (X.W.); lyliu@fudan.edu.cn (L.L.); 2 Department of Physics, Fudan University, Shanghai 200433, China

**Keywords:** whispering-gallery mode (WGM) microcavity, micro-bubble resonators, optomechanics, optical sensing and sensors, optical spring effect

## Abstract

In this work, we present a novel approach for obtaining the effective mass of mechanical vibration mode in micro-bubble resonators (MBRs). To be specific, the effective mass is deduced from the measurement of optical spring effect (OSE) in MBRs. This approach is demonstrated and applied to analyze the effective mass of hollow MBRs and liquid-filled MBRs, respectively. It is found that the liquid-filled MBRs has significantly stronger OSE and a less effective mass than hollow MBRs, both of the extraordinary behaviors can be beneficial for applications such as mass sensing. Larger OSE from higher order harmonics of the mechanical modes is also observed. Our work paves a way towards the developing of OSE-based high sensitive mass sensor in MBRs.

## 1. Introduction

Since the prediction from the theoretical work of Braginsky [1,2] that the mechanical vibrational oscillation and the optical mode in a Fabry-Pérot resonator can be coupled together, optomechanics has been investigated widely [3,4,5,6,7,8,9]. The optical force exerted on microcavity induces displacement of the cavity (breathing [9,10] or twisting [5,11] or stretching [12]). It was also found that when the displacement is large enough, it can also change the mechanical rigidity of material, thus varying the mechanical vibration frequency, named the optical spring effect (OSE) [13,14]. This effect has been first reported in Fabry-Pérot resonators [15,16], then an increasing number of works are focused on or related to this phenomenon in the solid photonic devices, such as photonic crystal cavities [5,6,11,17] and whispering-gallery mode (WGM) cavities [4,5,13,14]. On the other hand, the liquid and gas phases have great potential to exhibit distinguished optomechanical behaviors because of the significant difference in acoustical impedance. However, few works of cavity optomechanics have been done in non-solid phases of matter. Very recently, Yu et al. achieved single molecule sensing by OSE in a microsphere in aqueous environment [18].

Micro-bubble resonator (MBR) is a thin-wall glass cavity with a hollow tube for fluids. When the type of liquid in the tube is changed, it changes not only the optical field distribution of the optical resonant mode, but also the mechanical parameters of the cavity structure such as viscosity, sound speed and mass. Hence, MBR can easily bridge the cavity optomechanics and optofluidics by the microfluidic channels and a great deal of works come up [7,8,9,10,19,20,21]. The liquid filled in the MBR with a thin shell completely changes the whole mechanical system.

The effective mass of mechanical mode is an essential parameter for the characterization of the optomechanical mode. It can be used to calculate the zero point motion [12,22], the effective temperature [22,23], and other parameters [22,24]. Also, in the optomechanics mass sensing, the effective mass directly determines the detection limit of the sensor [25]. The effective mass can be significantly less than the real mass of a cavity, because only a part of the cavity is excited. Up to now, there are two ways to obtain the effective mass of a mechanical mode. One is a theoretical calculation from finite-element simulation [22]. The other is fitting the function of mechanical oscillation threshold with the Q of different optical modes [22]. While the former is limited by the actual shape of the cavity, the latter costs too much time in the testing of the mechanical oscillation threshold curve. 

In this work, a systematic investigation of the OSE in MBRs is reported. A new way to obtain the effective mass of mechanical modes from OSE measurements is developed. In particular, we found that when a MBR is filled with a kind of liquid, both OSE and effective mass have extraordinary behaviors.

## 2. Theory of Optical Spring Effect in WGM Microcavity

In the WGM microcavity, the small mode volume and high Q value can lead to high optical field intensity. The circling light in the cavity generates strong radiation pressure. Hence, the effective spring constant of the vibration mechanical eigenmode changes. This effect is named optical spring effect (OSE) or “light induced mechanical rigidity” [13,14,22]. The mechanical frequency shifted by OSE can be described as the following (assuming Ω*_m_* + Ω_0*m*_ ≈ 2Ω_0*m*_, here Ω_0*m*_ is the intrinsic angular frequency of the mechanical mode. In other words, Ω_0*m*_ is the vibration frequency without laser in the cavity. While, Ω*_m_* is the vibration frequency of the cavity when the light is in it [13,14].

(1) $$\begin{array}{ll} \Omega_{m}=2\pi f_{m} & =\sqrt{\frac{k_{0}+k_{os}}{m_{eff}}}\\ & =\sqrt{\frac{m_{eff}\Omega_{0m}^{2}+k_{os}}{m_{eff}}}\approx \Omega_{0}{}_{m}+\frac{k_{os}}{2\Omega_{0}{}_{m}m_{eff}}, \end{array}$$

in which *f_m_* and *m_eff_* are the mechanical frequency and the effective mass of the mechanical mode. The intrinsic mechanical spring coefficient *k*_0_ is determined by the structure, the material, and other physical and mechanical parameters of the MBR. In Equation (1), only the parameter *k_os_*, the optical spring coefficient, is related to the optical field. *k_os_* can be described by [13],

(2) $$\begin{array}{ll} k_{os}=-\frac{d}{dr}F_{rp}(r) & =-\frac{d}{dr}(\frac{2\pi n}{c}P_{circ})\\ & =\frac{2\omega_{0}^{2}}{R_{0}^{2}Q_{ext}}\frac{\Delta \omega }{{\left(\Delta \omega^{2}+\delta^{2}\right)}^{2}}P_{i}\\ & =\frac{-16\pi^{3}c^{3}\Delta \lambda }{R_{0}^{2}Q_{ext}}\frac{1}{{\left({\left(\frac{2\pi c\Delta \lambda }{\lambda_{0}{}^{2}}\right)}^{2}+\delta^{2}\right)}^{2}}P_{i}, \end{array}$$

in which *F_rp_*(*r*) is the radiation pressure, which is a function of radius. For the structural differences between MBRs and microtoroid cavities, there is no such an offset factor in the *F_rp_*(*r*) when compared with Ref. [14]. *n* is the effective refractive index of the optical mode. *R*_0_ is the radius of the microcavity. *c* is the speed of light in vacuum. *ω*_0_ and *λ_0_* are the resonant frequency and wavelength. *Q_ext_* is external optical quality factors and *δ* is the total linewidth of the microcavity in round frequency. The radiation pressure is related to the circulating light field. *P_circ_* is the optical power circling in the microcavity. *P_i_* is the input optical power. The optical frequency detuning is ∆*ω = ω_laser_ − ω_cavity_*. Equation (2) tells that *k_os_* changes linearly with *P_i_*, consequently Ω*_m_* changes (i.e., OSE). On the other hand, the mechanical vibration frequency varies with ∆*ω*. Hence, by fitting the curve of Ω*_m_* with detuning ∆*ω*, which can also use Δ*λ* to describe, it is possible to deduce the effective mass *m_eff_*, in the case that the other parameters in Equation (2) can be determined experimentally. 

## 3. Experiment and Result Analysis

**Figure 1:**
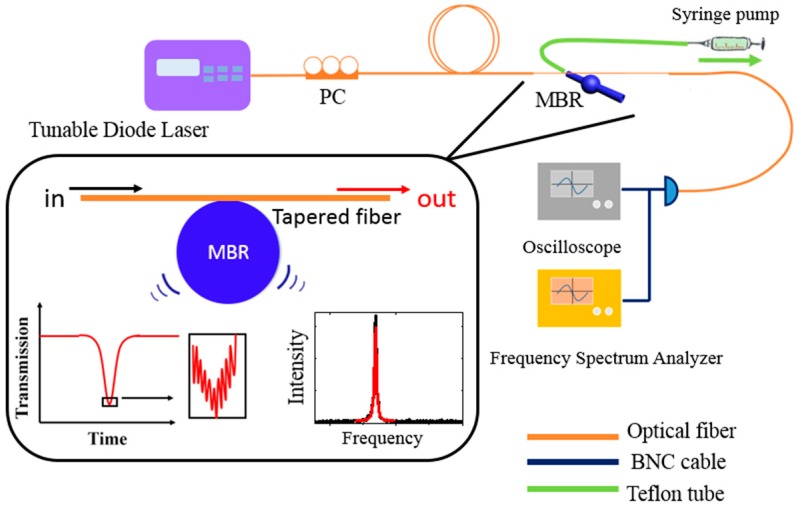
The experimental setup and illustration of the measuring system. Laser, from a tunable laser (Anritsu Tunics Plus CL), is coupled to a micro-bubble resonator (MBR) by a tapered fiber. Polarization controller (PC) in the path is used to control the polarization of the laser. A high speed photoelectric detector (PD) is employed to collect optical signals and convert optical signals into electrical signals. The electrical signals are divided into two ways to the oscilloscope and frequency spectrum analyzer by the BNC (Bayonet Neill-Concelman) cable. The transmission of the laser will contain the mechanical oscillation frequency and can be obtained by the frequency spectrum analyzer. The MBR samples are connected with a syringe pump for the liquid injection.

The MBR samples were prepared using quartz capillary tube by the fuse-and-blow technique [26]. MBRs with different wall thickness were obtained by thinning the quartz capillary tube wall in hydrofluoric acid solution before blowing. An experimental setup that was described in Ref. [10] was adopted for the OSE measurement (Figure 1). During the experiment, the wavelength of the pump laser from the tuning diode laser (Anritsu Tunics Plus CL, Atsugi-shi, Kanagawa, Japan) was changed gradually around 1.55 μm and the mechanical oscillation frequency of the MBR was measured by a spectrum analyzer (Agilent Technologies E4402B, Santa Clara, CA, USA).

**Figure 2:**
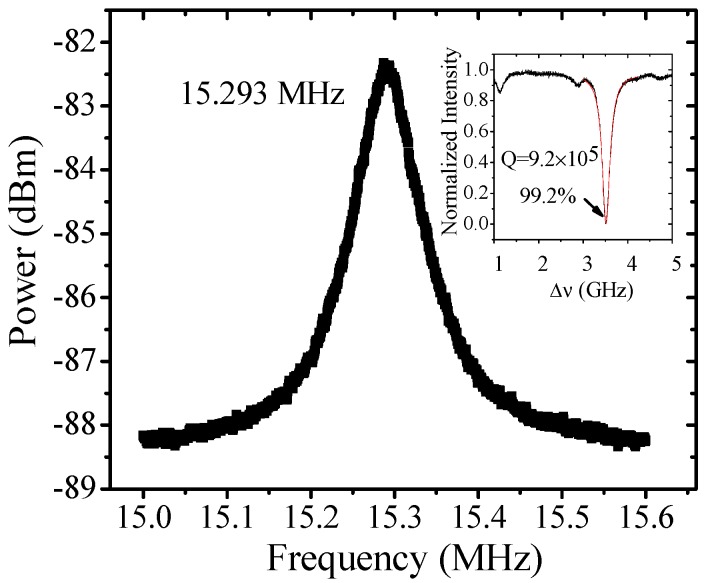
Mechanical spectra of a hollow micro-bubble resonator (MBR) (diameter 168 μm and wall thickness 5–6 μm) at 6.5 mW laser pump. Inset: The transmission spectrum of the optical resonant mode. The quality factor of the mode is 9.2 × 10^5^ measured at low laser power.

**Figure 3:**
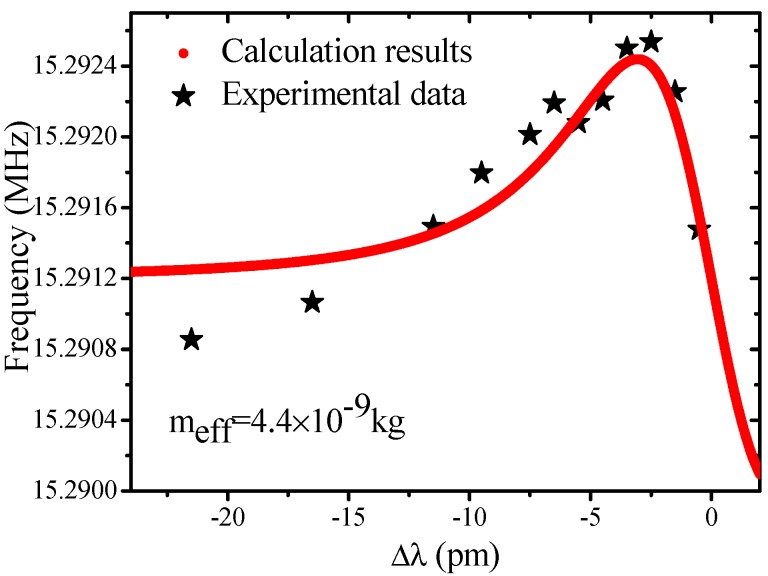
The optical spring effect (OSE) measured at 1 mW pumped power. Red lines are the simulated results with the effective mass of 4.4 × 10^−9^ kg and the experimental data. Δ*λ* = *λ_laser_* − *λ_cavity_*.

Figure 2 shows a typical mechanical spectrum of a MBR with 168 μm outer diameter and 5–6 μm wall thickness (estimating by an optical microscopy) at a pump power of 6.5 mW. As shown in the inset of Figure 2, the total quality factor of the optical mode is 9.2 × 10^5^. The normalized transmission is almost zero at the center of resonant mode, which indicates that the optical mode is under a critical-coupling regime. Figure 3 shows the measured mechanical oscillation frequency as a function of laser frequency detuning in the same MBR. By substituting the experimental data (*Q_tot_* = 9.2 × 10^5^, *Q_ext_* = 1.84 × 10^6^, outer radius of the MBR = 84 μm, net input power = 0.35 mW) into Equation (2), *m_eff_* of the mechanical mode can be deduced to be 4.4 × 10^−9^ kg. The greatest uncertainty in the measurement comes from the *Q_ext_*. According to the first-order error analysis, here, the *Q_ext_* brings the uncertainty of 9% in the measurement of *m_eff_*. Meanwhile, the actual mass of the microbubble sample is estimated to be 6.6 × 10^−9^ kg by using the concentric circle model mentioned in Ref. [27], which is the same order of magnitude when compared with the experimental result. The difference between *m_eff_* and actual mass can be explained by the following reasons. First, the *m_eff_* represents the moving mass of the mechanical mode, which is only a part of the actual mass [4]. From our experimental model, the effective mass accounts for more than 60% of the real mass. Which means that the moving mass in this mechanical mode is largely driven by the optical force. Second, the non-uniform shape of the actual sample is not considered in the model. The actual wall thickness is non-homogeneous in axial direction, while in the calculating model a uniform wall thickness has been used.

**Figure 4:**
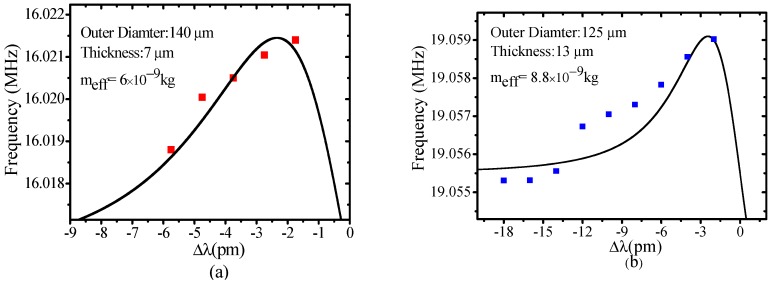
The OSE measurements of two MBRs with different wall thickness. (**a**) MBR outer diameter 140 μm and wall thickness 7 μm; (**b**) MBR diameter 125 μm and wall thickness 13 μm. Dots are the experimental results, and solid lines are simulated results. Effective masses of 6 × 10^−9^ kg and 8.8 × 10^−9^ kg are obtained.

The mechanical vibration frequency is significantly affected by the wall thickness of the MBR. A thinner wall MBR leads to a lower mechanical mode frequency [19,28]. Figure 4a,b show the OSE of two MBRs with a wall thickness of 7 μm and 13 μm, respectively. The MBRs were excited by optical modes with approximate equal *Q_tot_* (1.3 × 10^6^ and 1.5 × 10^6^), and the mechanical mode at 16.02 MHz and 19.056 MHz were monitored for OSE measurement. In the fitting process, the relationship of *Q_ext_* and *Q_tot_* is fixed to be *Q_ext_* = 3*Q_tot_* (in this work, all of the the coupling regimes are under coupled regimes which can be determined by the gap between the fiber and the microcavity). The effective mass of the two MBRs was deduced to be 6 × 10^−9^ kg and 8.8 × 10^−9^ kg, respectively. Correspondingly, the actual mass is 8.0 × 10^−9^ kg and 1.1 × 10^−8^ kg, respectively. The subtle difference in the effective mass of the two MBRs indicates that the moving mass excited by the optical mode in the two samples are very similar, because the moving mass is basically limited by the penetration depth of the radial optical mode.

**Figure 5:**
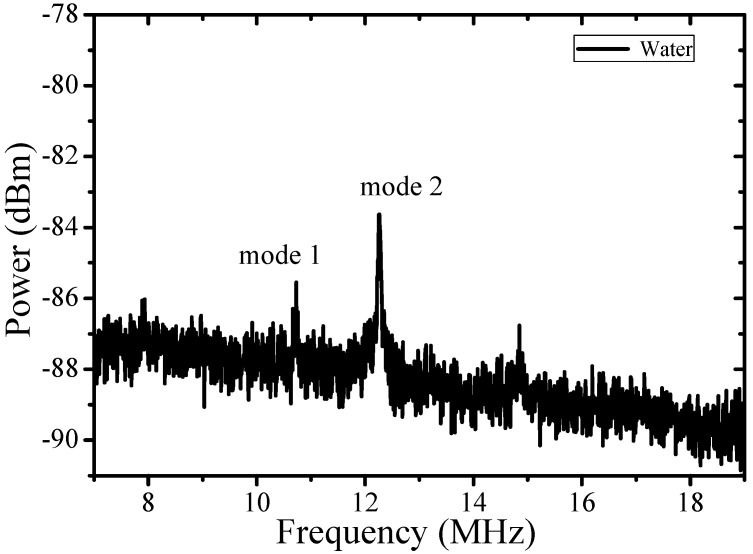
Measured mechanical mode spectrum of a MBR filled with water. Two mechanical modes have been observed in the spectrum named mode 1 and mode 2.

**Figure 6:**
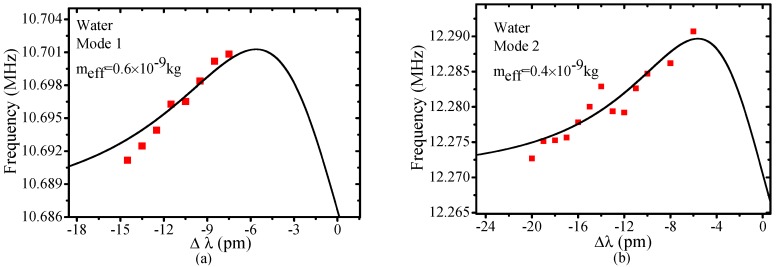
OSE of mode 1 (**a**) and mode 2 (**b**) as described in Figure 5. Solid red squares are the experimental data and the black lines are the simulated results, *m_eff_* of 0.6 × 10^−9^ kg and 0.4 × 10^−9^ kg are obtained.

The hollow structure of MBR provides a way to change the mechanical properties of the system by filling it with different liquids. Such a solid-liquid hybrid cavity largely changes the optical mode field distribution in thin wall cavities. Meanwhile, the mechanical response can be different even in relatively thick wall cavities, because the mechanical resonant wavelength is much longer. Therefore, the mechanical frequency and the effective mass of the system can be regulated drastically. Figure 5 is the mechanical spectrum of a water-filled MBR. The MBR has an outsider radius of 140 μm and wall thickness of 7 μm (the same sample as that in Figure 4a). There are two mechanical modes in a range of 10–12 MHz, excited by the same optical mode (*Q* = 5 × 10^5^). The OSE of the two modes are shown in Figure 6a,b. The effective mass of the two modes are 0.6 × 10^−9^ kg and 0.4 × 10^−9^ kg, respectively. Note that in water-filled MBR, mechanical mode frequency shifts toward lower frequency. However, the effective mass of the mode is nearly an order of magnitude less than that of the hollow MBR, which is extraordinary considering that the real mass of a liquid-filled bubble increases.

In the water-filled MBRs, the effective masses are changed greatly for the following reasons. First, the elasticity coefficient is mainly determined by the liquids in the solid-liquid hybrid mechanical system. The difference between the elasticity coefficient of solid and liquid can be one order of magnitude (i.e., *k*_liquid_ << *k*_solid_). Moreover, the density of liquid is much less than that of solid. Second, the testing mechanical modes when filled with liquids may be high order mechanical modes. The moving mass excited by the optical mode in the high order mechanical modes is less. Due to the small compressibility of liquid, there is big internal stress at the solid-liquid interface. Thereby, the vibration of solid spherical shell of the microbubble is limited. On the other hand, after the liquid is filled, the mechanical vibration in solid-liquid hybrid cavity can be equivalent to the vibration of the two series connected spring with very different elasticity coefficient. The final total elasticity coefficient can be expressed as following:

(3) $$k_{\mathrm{total}}=\frac{k_{\mathrm{solid}}\cdot k_{\mathrm{liquid}}}{k_{\mathrm{solid}}\;+\;k_{\mathrm{liquid}}}\approx k_{\mathrm{liquid}}$$

The elasticity coefficient of solid is much larger than the elastic coefficient of the liquid, so the elasticity coefficient of the total system is determined by the elasticity coefficient of the liquid.

**Figure 7:**
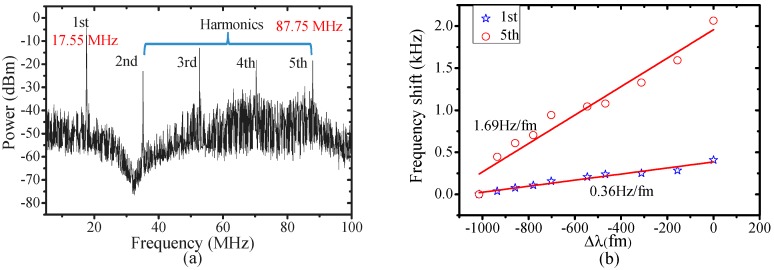
(**a**) Mechanical spectra of a hollow MBR above threshold power; (**b**) OSE of the 5th harmonic (hollow red circle) and the fundamental (hollow blue five-pointed star) modes.

Finally, the high harmonics of mechanical vibration can enhance OSE, which has been reported in many previous works [29]. The high harmonics can be generated by launching higher power into the cavity, which has been studied in [30]. Figure 7a shows the high harmonics spectrum of a MBR (outer diameter 120 μm, wall thickness 8 μm) pumped at 5.5 mW. The changes of fundamental and 5th harmonic mechanical mode frequency as a function of laser detuning are plotted in Figure 7b. The 5th harmonic mode frequency shifts are about 4.7 times larger than that of the fundamental mode when the pump laser detunes, which agrees with that reported results in [29] that the nth harmonic mechanical frequency movement is roughly *n* times of the fundamental frequency. The findings provide a new approach for increasing the mechanical mode sensitivity and enlarging the OSE.

## 4. Conclusions

In summary, we investigated the OSE in MBR and developed a new method for the calculation of effective mass. The effective mass of a low radial order breathing mechanical mode in MBR is around 10^−9^ kg, which is obviously less than that of the real cavity mass. Meanwhile, the effective mass is getting closer to the real cavity mass as the wall thickness decreases. The liquid-filled MBR has significantly less effective mass. Smaller effective mass means a higher sensitive mass detection, therefore, it is possible to select suitable liquid to achieve a better detection result for the OSE-based sensor. High harmonic mechanical modes can be also used to enlarge the OSE and thus increase the sensitivity in OSE sensing.
